# Inclusion of Red Macroalgae (*Asparagopsis taxiformis*) in Dairy Cow Diets Modulates Feed Intake, Chewing Activity and Estimated Saliva Secretion

**DOI:** 10.3390/ani13030489

**Published:** 2023-01-31

**Authors:** Emma Nyløy, Egil Prestløkken, Margrete Eknæs, Katrine Sømliøy Eikanger, Live Heldal Hagen, Alemayehu Kidane

**Affiliations:** 1 Department of Animal and Aquacultural Sciences, Faculty of Biosciences, Norwegian University of Life Sciences, 1430 Ås, Norway; 2 Faculty of Chemistry, Biotechnology and Food Science, Norwegian University of Life Sciences, 1430 Ås, Norway

**Keywords:** seaweed, animal behaviour, dairy cows, methane

## Abstract

**Simple Summary:**

Here, we studied how *Asparagopsis taxiformis*—an emerging ruminant feed additive with methane inhibitory effect—affected the feed intake of early lactating dairy cows given free access to total mixed ration and drinking water. Eating–rumination behaviour was recorded continuously for 11 days, using electronic sensors, on 15 lactating Norwegian Red dairy cows fed diets with graded levels (0, 0.125 and 0.25% on an organic matter basis) of the macroalgae. Our results indicated that the macroalgae reduced feed intake through reduced feed intake rate, and increased eating and chewing indices. Inclusion of the macroalgae increased the estimated saliva production per unit dry matter intake. To our knowledge, this is the first documentation on the eating–rumination behaviour of dairy cows given access to a diet containing this novel ingredient. Alternative modes of delivery of the ingredient, in contrast to what is commonly used now (i.e., blending it with a water–molasses mixture) is envisaged.

**Abstract:**

The current study assessed the effects of red macroalgae *Asparagopsis taxiformis* (AT)—included as an enteric methane inhibitor—in dairy cow diets on feed intake and eating–rumination behaviour. Fifteen early lactating Norwegian Red dairy cows were offered ad libitum access to drinking water and a total mixed ration (TMR) composed of 35% concentrate feed and 65% grass silage on a dry matter (DM) basis. The experiment lasted for 74 days with the first 22 days on a common diet used as the covariate period. At the end of the covariate period, the cows were randomly allocated into one of three dietary treatments: namely, 0% AT (control), 0.125% AT and 0.25% AT in the TMR. The TMR was offered in individual feed troughs with AT blended in a 400 g (*w*/*w*) water–molasses mixture. Eating–rumination behaviour was recorded for 11 days using RumiWatchSystem after feeding the experimental diets for 30 days. The 0.25% AT inclusion significantly reduced the DM intake (DMI). Time (min/d) spent on eating and eating in a head-down position increased with the increasing AT level in the diet, whereas rumination time was not affected. The greater time spent on eating head-down with the 0.25% AT group resulted in a significantly higher chewing index (min/kg DMI). Estimated saliva production per unit DMI (L/kg DMI, SE) increased from 10.9 (0.4) in the control to 11.3 (0.3) and 13.0 (0.3) in the 0.125% and 0.25% AT groups, respectively. This aligned with the measured ruminal fluid pH (6.09, 6.14, and 6.37 in the control, 0.125% AT and 0.25% AT groups, respectively). In conclusion, either the level of the water–molasses mixture used was not sufficient to mask the taste of AT, or the cows used it as a cue to sort out the AT. Studies with relatively larger numbers of animals and longer adaptation periods than what we used here, with varied modes of delivery of the seaweed may provide novel strategies for administering the additive in ruminant diets.

## 1. Introduction

Dry matter intake (DMI) is the most important parameter determining the performance of dairy cows. Behavioural assessments, such as eating–rumination activities, are often used to study production parameters related to the DMI, intake pattern and feed efficiency [1,2,3,4]. Furthermore, such behavioural assessments, along with DMI, are important non-invasive measurable parameters of ruminant health [5]. 

Animals behave differently when faced with changes in the diet [6]. Consequently, any non-conventional feed ingredient might elicit behavioural changes with consequences on intake, health and performance. Efforts geared towards sustainable food production, reduced environmental footprints of animal production and improved production efficiency necessitated the use of alternative non-conventional feed resources and feed additives, especially in ruminant diets [7,8,9,10]. Feed additives, such as 3-nitrooxypropanol (3-NOP), lipids and red seaweed (e.g., *Asparagopsis taxiformis* or *Asparagopsis armata*) are some of the common ones in ruminant diets gaining interest for their purported inhibitory effect on enteric methane emissions [7,8,10,11,12]. Some of these additives (e.g., 3-NOP) are reported to reduce methane without significant decrease on DMI [12,13], whereas the inclusion of *Asparagopsis* spp. in ruminant diets consistently affected feed intake and performance [7,14] and, to some degree, gut health [8,14]. For instance, at a 1% inclusion level (on an organic matter basis), *Asparagopsis armata* reduced DMI by about 38%, relative to the control diet in dairy cows [7], whereas *Asparagopsis taxiformis* (AT), at a 0.5% inclusion level, reduced DMI by about 18% in high and medium forage diets of beef steers [11]. In addition to the DMI, knowledge of time cows spend chewing or ruminating is regarded as a valuable management tool in terms of optimizing cow health [15]. Despite the increased interest of *Asparagopsis* spp. as a methane reducing agent, the effect of its inclusion in dairy cow diets on feed intake pattern and eating–rumination behaviour has, to the best of our knowledge, not been reported before. 

Therefore, the objective of this study was to assess the effects of AT inclusion on feed and water intake, and the eating–rumination behaviour of Norwegian Red dairy (NRF) cows given ad libitum access to total mixed ration (TMR) containing graded levels of AT and drinking water. We hypothesized that the delivery of AT with a water–molasses mixture in the TMR to dairy cows will mask the taste of AT. Consequently, the AT, at the levels included here, will not affect the eating–rumination behaviour, chewing indices and DMI. 

## 2. Materials and Methods

This experiment was conducted from early-March to end of May 2021, at the Metabolism Unit of the Department of Animal and Aquacultural Sciences at the Norwegian University of Life Sciences, Norway.

### 2.1. Animals and Experimental Design

Fifteen lactating NRF cows (i.e., 6 intact and 9 rumen canulated) in their 2nd to 4th parity with mean (±SD) initial body weight of 681 (±56.1) kg, daily milk yield (MY) of 36.9 (±4.2) kg and days in milk (*DIM*) of 95 (±25.8) were used in the experiment. The experiment lasted for 74 days, including 22 days on a common diet as a covariate period and additional 13 days on adaptation to the experimental diets. The remaining 39 days feeding was designated as the experimental period. At the end of the covariate period, the cows were grouped into 3 based on the covariate period feed intake, MY, parity (2nd or older lactation) after blocking for canulation status (i.e., 3 rumen canulated and 2 intact cows per group). The groups were then randomly allocated to the dietary treatments prepared as TMR containing 0% (control), 0.125% (0.125% AT), and 0.25% (0.25% AT), on an organic matter basis using a randomized block design.

The animals were kept in a tie-stall accommodation with individual feed troughs, and free access to gauged waterlines with daily individual electronic registration of water intake. The cows were milked twice daily using a DeLaval milking machine (Delaval DelPro MU480; DeLaval Inc., Tumba, Sweden) between 07.00 and 08.00 (morning milking) and between 19.00 and 20.00 (evening milking).

### 2.2. Feeds and Feeding

A TMR composed of 65% (on DM basis) early 1st cut grass silage and 35% commercial concentrate feed was prepared at least 3 times per week. The TMR was mixed using a Siloking Duo 1814 (Kverneland, Bryne, Norway) mixing machine with about 20 min of chopping the grass silage followed by 10 min of mixing the chopped silage and concentrate feed. The grass silage was prepared from timothy (*Phleum pratense*) based stand of a 3rd year ley seeded as SPIRE Surfôr/Beite Pluss 10 (Felleskjøpet Rogaland Agder, Stavanger, Norway) containing 42% timothy, 23% meadow fescue, 15% blue grass, 10% white clover and 10% perennial ryegrass. The commercial concentrate feed (i.e., Drøv Energirik Høg) was manufactured by Norgesfôr (Norgesfôr AS, Oslo, Norway) suited for high yielding NRF dairy cows (i.e., 7000–10,000 kg energy corrected milk per lactation; https://www.norgesfor.no/strand-unikorn/produkt/drov-energirik/; accessed on 15 November 2022).

During the feeding period, the cows were given ad libitum access to a TMR delivered in three split portions, i.e., 40%, 30% and 30% of their daily allowance at 07.15, 14.15 and 19.15 h local time, in respective order. The TMR was offered in individual feed troughs with collection of daily orts just before the 07.15-h feeding. 

Freeze-dried and crushed AT was blended with 400 mL of a sugar beet molasses and water mixture (50:50 *w*/*w*). For the 0.125% AT and 0.25% AT cows, the AT–water–molasses mixture was hand mixed with the TMR in individual feed troughs with amounts proportional to their DM offer. The control group was given the same amount of molasses–water mixture to avoid any distortion in chemical composition of the feed offered. 

The cows had free access to their feed except for a brief moment when feed refuse was collected and when the AT and water–molasse mixture was hand mixed at the three feed delivery times. Areas around the feed troughs were maintained clean and feed tossed from the troughs, whenever this happened, was put back during any given time of the day. 

### 2.3. Feed Samples, Analysis and Chemical Composition

Duplicate TMR samples were taken from each mixing day to monitor the planned DM content and to estimate the DMI of the cows. The samples were dried at 60 °C for 48 h and DM content was estimated with adjustment for volatile component loss, according to the Nordic feed evaluation system [16]. 

Representative grab samples of the TMR offered and the TMR refused were taken in duplicate once a week and kept frozen at −20 °C until the end of the experiment. Later, the weekly samples were pooled, freeze-dried, and milled using a cutting mill (Restch SM 200, Retsch GmbH, Haan, Germany) with a 1.0 mm sieve size for chemical analysis, except for starch which required a 0.5 mm sieve size. The samples were analyzed for neutral detergent fibre corrected for residual ash (NDFom), acid detergent fibre corrected for residual ash content (ADFom), dietary nitrogen (to estimate crude protein i.e., N × 6.25), starch, crude fat and ash content, as recently described in Kidane et al. [17]. The sum of the residual carbohydrates and silage fermentation products in the TMR was calculated as 1000 minus the sum of ash, NDFom, CP, starch and crude fat, all expressed in g per kg DM. The summarized results on the chemical composition are provided in Table 1.

### 2.4. Eating, Rumination Activities and Ruminal Fluid pH

Eating–rumination behaviour was recorded for 11 days after feeding the experimental diets for 30 days. During the recording period, all cows were fitted with RumiWatch noseband sensors (NBS) from the RumiWatchSystem (ITIN + HOCH GmbH, Liestal, Switzerland; developed by AGROSCOPE, in collaboration with ITIN + HOCH GmbH and VETSUISSE; Switzerland) (https://www.rumiwatch.com; accessed on 15 November 2022). Details of this system and method of operations with additional specifications have been presented in previous studies [5,18]. The NBS has a pressure sensor integrated in the noseband of a halter, recording jaw movements and matching them to activities, such as eating (i.e., eating head-up position, and eating head-down position), ruminating, drinking and other activities [3,5,19]. In addition, data on the total eating chews (eating head-down, i.e., feed selection, acquisition and chewing; and eating head-up, i.e., mastication of the acquired feed and creating a bolus before swallowing) and total rumination chews were produced. At the end of recording, the data were downloaded to a computer and converted to a CSV format using RumiWatch Converter software V0.7.4.13 (FW00.62). The data were split into 24 h intervals using 1 h resolution. The pH of the ruminal fluid samples taken on three sampling days during the 39 experimental days (from the central rumen through rumen canula for cannulated cows, and through oesophageal tubing for intact cows) was measured using portable pH meter (pH 3310) (WTW GmbH, Weilheim, Germany) fitted with a Polyplast Pro sensor (Hamilton, Bonaduz AG, Switzerland).

### 2.5. Calculations, Data Summary and Statistical Analyses

Chewing data collected over the recording period was summed up in daily time budgets (min/d). Furthermore, to provide an insight into the diurnal pattern, as influenced by the AT inclusion and feed delivery times over the recording days, behavioural activities were presented with data split into 24 h real time (min/h) of the behavioural activities. Two of the recording devices (one in the control group and another in the 0.25% AT group) failed to record and some of the units stopped recording towards the end of the recording period due to battery depletion, resulting in a total of about 3000 h of recording shared among 13 cows.

The dry matter intake rate was calculated for each cow based on the recorded amount of DM consumed, and time spent on eating (sum of head-up and head-down eating time) (Equation (1)).

(1) $$\mathrm{DMI}\;\mathrm{rate},\;g/\min=\frac{\mathrm{Daily}\;\mathrm{DMI}\;\left(g/d\right)}{\mathrm{Time}\;\mathrm{spent}\;\mathrm{on}\;\mathrm{eating}\;\left(\min/d\right)}$$

The eating index (EI, min/kg DMI) was calculated as the total time spent on eating (min/d) divided by the total DMI (kg/d) (Equation (2)), whereas rumination index (RI, min/kg DMI) was calculated as total time spent on rumination (min/d) divided by the total DMI (kg/d) (Equation (3)).

(2) $$\mathrm{EI},\;\min/\mathrm{kg}\;\mathrm{DMI}\;=\frac{\mathrm{Time}\;\mathrm{spent}\;\mathrm{on}\;\mathrm{eating}\;\left(\min/d\right)}{\mathrm{Daily}\;\mathrm{DMI}\;\left(\mathrm{kg}/d\right)}$$

(3) $$\mathrm{RI},\;\min/\mathrm{kg}\;\mathrm{DMI}\;=\frac{\mathrm{Time}\;\mathrm{spent}\;\mathrm{on}\;\mathrm{rumination}\;\left(\min/d\right)}{\mathrm{Daily}\;\mathrm{DMI}\;\left(\mathrm{kg}/d\right)}$$

The chewing index (CI, min/kg DMI), was calculated as the total time spent on eating plus rumination divided by the total DMI (Equation (4)), whereas the number of chews per unit DMI was calculated as the total eating and ruminating chews (counts/d) divided by the daily DMI (Equation (5)).

(4) $$\mathrm{CI},\;\min/\mathrm{kg}\;\mathrm{DMI}=\frac{\mathrm{Time}\;\mathrm{spent}\;\mathrm{on}\;\mathrm{eating}+\mathrm{rumination}\;\left(\min/d\right)}{\mathrm{Daily}\;\mathrm{DMI}\;\left(\mathrm{kg}/d\right)}$$

(5) $$\mathrm{Chews}\;\mathrm{per}\;\mathrm{unit}\;\mathrm{DMI},\;\mathrm{counts}/\mathrm{kg}\;=\frac{\mathrm{Eating}+\;\mathrm{rumination}\;\mathrm{chews}\;\left(\mathrm{counts}/d\right)}{\mathrm{Daily}\;\mathrm{DMI}\;\left(\mathrm{kg}/d\right)}$$

The daily saliva production was estimated assuming a fixed rate of saliva secretion per minute of eating (0.225 L/min), ruminating (0.225 L/min) and resting (0.114 L/min), as reported by Maekawa M, Beauchemin KA and Christensen DA [20] and Bailey CB [21]. The saliva volume per unit DM consumed (L/kg DMI) was calculated as the total estimated daily saliva secretion (L/d) divided by DMI.

The data collected over the recording days were analyzed with SAS statistical package (SAS for Windows, SAS version 9.4) as repeated measurements with Proc Mixed using an autoregressive (AR1) covariance structure with a cow as repeated subject. The number of chews per unit DMI (count data) was log transformed (log10 _(count+1)_) before the statistical analysis, to stabilize the variance. Data summarized as time spent on activities per day (min/day) were analyzed using the following model:

*Y_ijk_* = *μ* + *Diet_i_* + *Day_j_* + *P_k_* + (*Diet* × *Day*)*_ij_* + *DIM* + *e_ijk_*

where *Y_ijk_* = the recorded variable (e.g., eating time, min/day); *μ* = overall mean; *Diet_i_* = fixed effect of AT inclusion level (*i* = 0, 0.125 and 0.25% AT); *Day_j_* = effect of recording day (*j* = 1, 2, 3 …, 11); *P_k_* = fixed effect of parity group (l = 2nd lactation and 2 = older lactation); (*Diet* × *Day*)*_ij_*, = interaction effect of AT inclusion level and day of recording; *DIM* is the fixed effect of the stage of lactation expressed as days in milk at the start of the experiment; *e_ijk_* = residual error. 

Similarly, other daily recorded (e.g., DMI, drinking water intake) and estimated parameters (e.g., CI, EI, RI, DMI rate, chews per minute) were analyzed using the above model. In the results, least square means with S.E. are presented with the statistical significance declared at *p* ≲ 0.05 and tendencies discussed with 0.05 < *p* < 0.1. 

## 3. Results and Discussion

### 3.1. Feed and Water Intake

Data on the DMI and freshwater intake (FWI) over the behavioural recording days is presented in Table 2. During the eating–rumination behaviour recording period, all groups showed stable intake of both feed and drinking water. However, the DMI was significantly lower in the 0.25% AT group, compared to the other two groups. Recent experiments involving the macroalgae with dairy cows [7,9,14] and beef steers [11] also reported a reduction in the DMI at varying inclusion levels. The underlying mechanisms for this reduced DMI are still elusive, but taste and post-ingestive negative controls from the dietary components in the macroalgae could partly explain as the animals learn to associate the flavor of food (i.e., taste, smell and texture) with the food’s post-ingestive consequence [22]. However, separate feeding of AT and a basal diet did not avoid a reduction in the basal feed intake in lactating dairy cows [13], suggesting an additional mechanism of feed aversion due to the AT consumption. Thus, the reduced DMI could also be due to an increased rumen hydrogen (H_2_) partial pressure caused by its low turnover to CH_4_ [23], even though the experimental results are inconsistent. On the contrary to the DMI, the FWI did not differ among the dietary treatments. The observed FWI was positively correlated (R = 0.668; *p* < 0.0001) with the DMI over the recording days (Supplementary Figure S1). A simple linear regression showed that cows consumed 3.61 (SE 0.33) L of water per kg DMI. This value is closely comparable to what was reported for Friesian cows (3.7 kg water/kg DMI) fed diets containing various forages [24]. The observed correlation agrees with other reports for dairy cows getting ad libitum access to feed and drinking water [25,26,27]. 

### 3.2. Time Budget on Eating, Rumination and Other Activities

**Figure 1:**
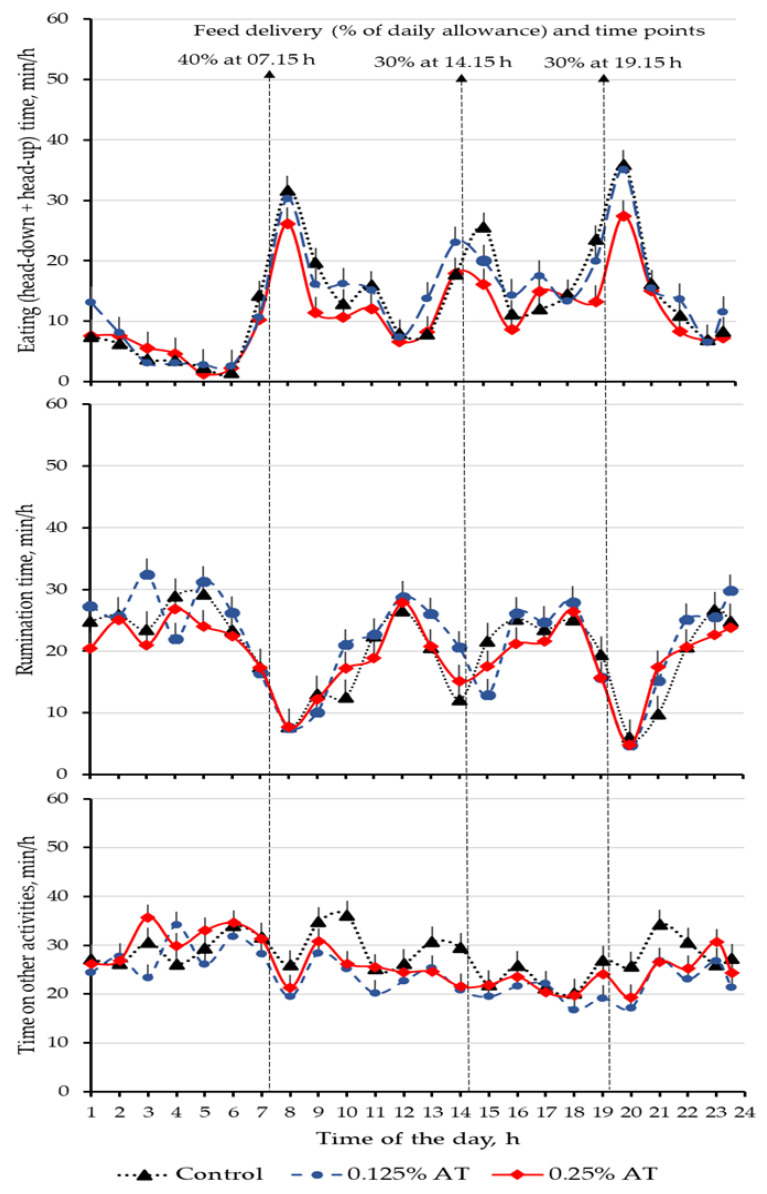
Diurnal fluctuation in eating, rumination and resting behaviours of Norwegian Red dairy cows offered ad libitum access to drinking water and total mixed ration with graded levels of *A. taxiformis*.

Data on the eating–rumination behaviour is presented in Table 2, with diurnal fluctuation in eating–rumination behaviour presented in Figure 1. Overall, the daily time allocation (min/d) for eating in the head-up position, rumination and other activities was not different among the dietary treatments. The observed eating times in our study were comparable with previous reports for indoor fed dairy cows with comparable body weight (e.g., 600 kg) and consuming over 20 kg DM/day [28,29,30,31], but considerably lower than NRF cows grazing on summer pasture [3]. Notably, daily eating time with the head-down position linearly increased (*p* = 0.009) with the increasing AT inclusion level from 0 to 0.25%, with the total eating time (summed up as eating head-up and eating head-down; *p* = 0.042) showing a similar trend. These observed differences were against the lower DMI in the 0.25% AT inclusion level. The observed greater eating time in the 0.25% AT group could be due to increased feed searching as manifested by eating in the head-down position (control < 0.125% AT < 0.25% AT; following linear trend, *p* = 0.009). 

### 3.3. Chewing Index and Estimated Saliva Secretion

Data on the eating rate, chewing indices and saliva production are presented in Table 3. The DMI rate was significantly reduced with the AT inclusion in the TMR (*p* < 0.001) with a trend for a quadratic response (*p* = 0.06). This reduced DMI rate for the AT group (~65 g DM/min) was comparable to reported intake rate (73 g DM/min) for lactating Holstein cows fed a TMR composed of 60% silage and 40% concentrate [20]. Indeed, the estimate for the control group (i.e., 103.3 g DM/min) appeared much higher than what is reported by Maekawa M, Beauchemin KA and Christensen DA [20] for Holstein cows. Factors related to diet (i.e., ingredients and chemical composition) and animal (e.g., stage of lactation, intake capacity), and management (e.g., frequency of feeding) could partly explain the discrepancies. The calculated indices on eating, rumination and chewing increased with the increasing AT inclusion level following a linear trend (*p* < 0.01). Thus, the calculated total saliva secretion (L/d) and saliva secreted relative to the DMI (L/kg DMI) linearly increased with the increasing AT inclusion level (Table 3). Cows fed the AT diets tended to have a higher daily saliva production (*p* = 0.058), but the 0.25% AT inclusion induced a greater saliva production per unit DMI (*p* = 0.0001). Increased chewing activities may explain more saliva secretion per unit DMI at 0.25% AT. Even though hand mixing the AT–water–molasses mixture with the TMR in the feed troughs for individual cow was deemed ideal, visual assessment indicated the affinity of AT–water–molasses towards the concentrate part of the TMR. We speculate that the animals in the AT groups used the water–molasses mixture as a cue to sort out the AT. As a result, these animals would have consumed a diet with a higher proportion of grass silage than planned. In dairy cows fed diets offered as TMR varying in the forage to concentrate ratio (F:C; 0.4:0.6, 0.5:0.5 and 0.6:0.4) the amount of silage in the TMR had no effect on time spent eating per kilogram of DM, but the rumination index (min/kg DMI) increased linearly with the increasing F:C ratio [20]. Therefore, sorting against the concentrate (i.e., in favor of grass silage) by our AT cows would increase the fibre content of their diet with consequences on eating, rumination and chewing indices—affecting the salivary secretion [20,32]. 

A proxy for any feed sorting behaviour, beyond visual assessment, could have been an internal marker of the TMR sensitive enough to pick up the phenomenon. The starch content in the TMR offered was 134.5 g/kg DM (same TMR offered for all cows), whereas the starch content (g/kg DM) of the TMR refused by the control, 0.125% AT and 0.25% AT groups was 114, 141 and 146, in respective order. Since starch is contributed by the concentrate part of the TMR, any selection against the concentrate part of the TMR would enrich the starch content of the refused diet, and vice versa. Even though feeding TMR is aimed at balancing nutrient supply on a temporal scale [33] and reduced feed sorting [34], dairy cattle could still sort for or against feed components [35,36,37]. In our study, the starch content of the TMR offered and TMR refused by individual cows supported this feed sorting hypothesis.

Promoting chewing activities increases the salivary secretion of dairy cows [15], which helps reduce the risk of acidosis through the buffering effect of saliva, and may optimize fibre digestion [38,39]. Here, we observed increased chewing activities per kg DMI by cows supplemented with AT, leading to a higher estimated saliva secretion per kg DMI. In addition to this, astringency of the AT, in amounts consumed here, could be another factor influencing the saliva secretion and rumen buffering. To this end, the measured ruminal fluid pH fell within the optimal range (i.e., 6.0–7.0 [40]) but the 0.25% AT supplementation significantly increased the ruminal fluid pH, compared to the other treatments. The pH values aligned with the estimated saliva secretion, especially with saliva secreted per kg DMI. Even though the level of the AT inclusion here was very low, our data suggested that this macroalgae is potent in modulating the eating–rumination behaviour, saliva secretion and feed intake in dairy cows.

## 4. Conclusions

Our results indicated that 400 g molasses–water mixture was not sufficient to mask the taste of AT at the 0.25% inclusion level, and the DMI was reduced at this inclusion level. Cows probably used the AT water–molasses mixture as a cue for sorting against the AT. This was clearly indicated by the calculated indices of the intake parameters and time spent eating head-down in the feed troughs. Since both feed intake and nutrients therein are the main factors driving animal performance, reduced feed intake and lack of balance of nutrients in the consumed feed due to feed sorting, as modulated by the macroalgae, may have implications on nutrient use efficiency and animal performance. Studies with longer adaptation periods involving a larger number of animals along with varied modes of delivery of the seaweed may provide novel strategies for administering the additive to reduce enteric methane emissions from dairy cows while maintaining feed intake and animal productivity.

## Figures and Tables

**Table 1:** Chemical composition of the total mixed ration and *Asparagopsis taxiformis* (g/kg DM, unless otherwise mentioned).

Item	Total Mixed Ration ^1^	*Asparagopsis taxiformis*
DM content (g/kg feed)	360.0	950.0
Organic matter	922.9	483.0
Ash	77.1	517.0
Crude protein	178.1	na
NDFom ^2^	371.7	na
ADFom ^3^	196.8	na
Starch	134.5	na
Crude fat	39.1	na
RestCHO + FFP ^4^	199.5	na
NEL_20_ (MJ/kg DM) ^5^	6.63	na

^1^ Total mixed ration offered to cows during the behavioural recording period; ^2^ neutral detergent fibre of the total mixed ration corrected for residual ash; ^3^ acid detergent fibre of the total mixed ration corrected for residual ash; ^4^ the estimated sum of residual carbohydrates and silage fermentation products; ^5^ estimated net energy lactation at 20 kg DM intake calculated according to the Nordic feed evaluation system [16] with the grass silage to concentrate feed ratio of 0.65:0.35; na = not analyzed/not available.

**Table 2:** Dry matter and water intake and daily time budget for activities by Norwegian Red dairy cows offered ad libitum access to drinking water and total mixed ration with graded levels of *Asparagopsis taxiformis*.

Parameters	AT Inclusion (%, OM Basis)			Statistics		Contrasts ^1^	
Intake	0	0.125	0.25	SE	*p*-Value	*L*	*Q*
Dry matter intake, kg/d	22.1 ^b^	22.4 ^b^	19.9 ^a^	0.53	0.009	0.017	0.043
Water intake, kg/d	70.1	77.5	65.9	4.67	0.190	0.466	0.121
**Time Budget, min/d**							
Eating, head-up	139.1	138.0	121.5	24.1	0.873	0.636	0.78
Eating, head-down	90.5 ^a^	162.0 ^b^	246.2 ^c^	26.8	0.021	0.009	0.836
Sum eating (head-up + head-down)	229.5	301.4	367.4	36.4	0.092	0.042	0.944
Rumination	474.1	517.4	488.6	32.1	0.516	0.770	0.332
Other activities	688.8	596.2	567.9	59.3	0.317	0.221	0.623

^1^ Contrasts; *L* = linear; *Q* = quadratic. Means in a row with different superscripts are different at *p* < 0.05.

**Table 3:** Calculated saliva production and chewing indices of Norwegian Red dairy cows offered ad libitum access to drinking water and total mixed ration with graded levels of *A. taxiformis*.

Parameters	AT Inclusion (%, OM Basis)			Statistics		Contrasts ^1^	
	0	0.125	0.25	SE	*p*-Value	*L*	*Q*
DMI intake rate, g/min	103.3 ^b^	67.0 ^a^	62.4 ^a^	5.13	<0.001	<0.001	0.06
EI, min/kg DMI	10.7 ^a^	14.2 ^ab^	17.3 ^c^	1.38	0.031	0.011	0.90
RI, min/kg DMI	21.7 ^a^	22.8 ^ab^	24.0 ^b^	0.53	0.018	0.006	0.99
CI, min/kg DMI	32.1 ^a^	37.6 ^b^	41.3 ^c^	0.99	<0.001	<0.001	0.42
Chews, counts/kg DMI	1922 ^a^	2322 ^b^	2608 ^b^	177	0.0016	0.0005	0.22
Saliva secretion, L/d	236	255	254	7.10	0.058	0.033	0.11
Saliva per kg DMI, L	10.7 ^a^	11.4 ^a^	13.0 ^b^	0.38	0.0001	<0.001	0.14
Ruminal fluid pH	6.09 ^a^	6.14 ^a^	6.37 ^b^	0.064	0.028	0.012	0.62

^1^ Contrasts; *L* = linear; *Q* = quadratic; DMI, dry matter intake; EI, eating index; RI, rumination index; CI, chewing index; Means in a row with different superscripts are different at *p* < 0.05.

## Data Availability

The data presented in this study are available on request from the corresponding author.

## Supplementary Materials

The following supporting information can be downloaded at: https://www.mdpi.com/article/10.3390/ani13030489/s1, Figure S1: The relationship between recorded freshwater intake and daily dry matter intake by early lactating Norwegian Red dairy cows given ad libitum access to total mixed ration and drinking water.
