# Supplementary material for: Inclusion of Red Macroalgae (Asparagopsis taxiformis) in Dairy Cow Diets Modulates Feed Intake, Chewing Activity and Estimated Saliva Secretion
Source: Animals (Basel). 2023 Jan 31;13(3):489. doi: 10.3390/ani13030489 (PMC9913350; doi:10.3390/ani13030489)

Supplementary figure legend:

Figure S1:

The relationship between recorded freshwater intake and daily dry matter intake by early lactation Norwegian red dairy cows given *ad libitum* access to total mixed ration and drinking water (Solid common regression line, FWI (L/d) = 0.58 + 3.61*DMI (kg/d), Root MSE = 9.71; $R^2 = 0.45$, $P < 0.0001$; dark triangle = Control; blue circle = 0.125% AT, and red diamond = 0.25% AT).

Figure S1.

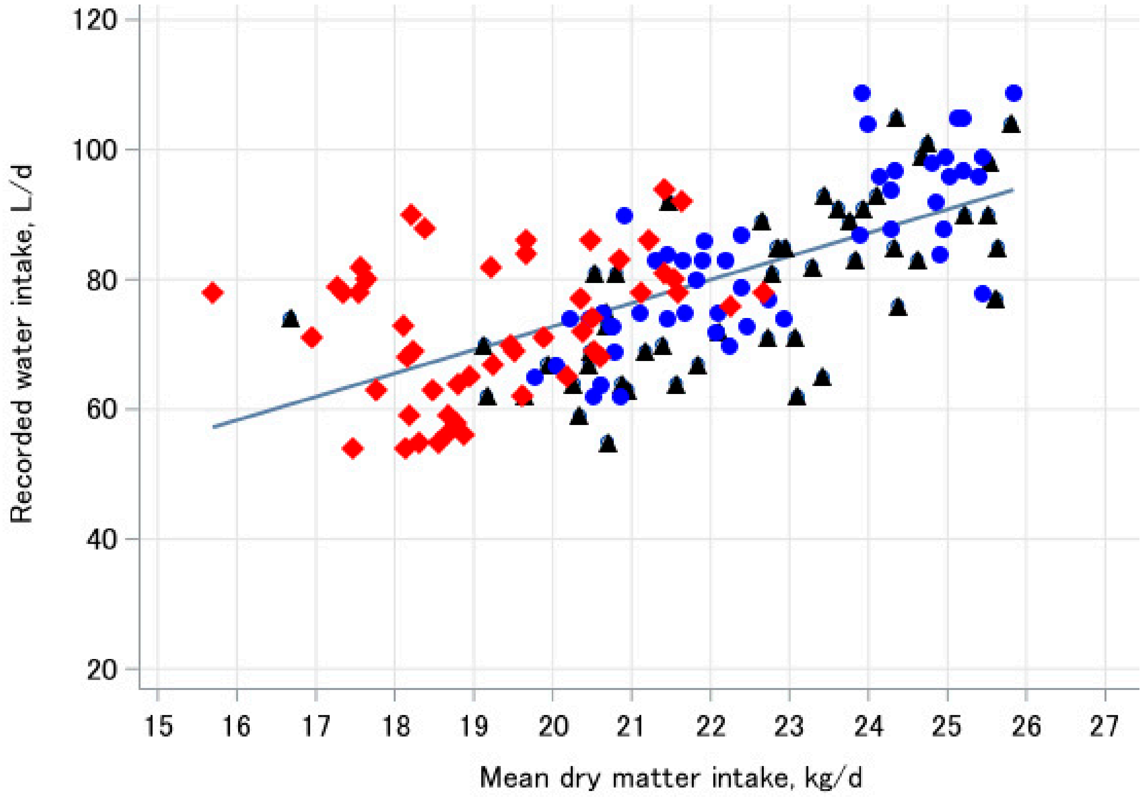

Supplement: Supplementary file 1 [file animals-13-00489-s001.zip › animals-2144954-supplementary.pdf]
